# Socio‐Organisational Aspects Related to Digital Competence Development Among Healthcare Professionals: A Cross‐Sectional Study With Cluster Analysis

**DOI:** 10.1155/jonm/4368819

**Published:** 2026-11-03

**Authors:** Janne Kommusaar, Laura-Maria Peltonen, Kadri Suija, Erika Jarva, Kristina Mikkonen, Lauri Sillanmäki, Mari Kangasniemi

**Affiliations:** ^1^ Department of Nursing Science Faculty of Medicine University of Turku Medisiina B Turku FI-20014 Finland utu.fi; ^2^ Faculty of Medicine Institute of Family Medicine and Public Health University of Tartu Ravila 19 Tartu 50411 Estonia ut.ee; ^3^ Department of Health and Social Management Faculty of Social Sciences and Business Studies University of Eastern Finland P.O. Box: 1627 Kuopio FI-70211 Finland uef.fi; ^4^ The Wellbeing Services County of Southwest Finland Turku University Hospital Kiinamyllynkatu 4-8 Turku 20520 Finland vsshp.fi; ^5^ Faculty of Health Sciences School of Medicine Institute of Public Health and Clinical Nutrition University of Eastern Finland P.O. Box: 1627 Kuopio FI-70211 Finland uef.fi; ^6^ Faculty of Medicine Research Unit of Health Sciences and Technology University of Oulu P.O. Box: 8000 Oulu FI-90014 Finland oulu.fi; ^7^ Medical Research Center Oulu Oulu University Hospital University of Oulu P.O. Box: 8000 Oulu FI-90014 Finland oulu.fi; ^8^ Department of Biostatistics University of Turku Turku FI-20014 Finland utu.fi

## Abstract

**Background:**

The increasing integration of digital technologies into healthcare has changed competence requirements for healthcare professionals, making digital competence essential for safe and effective care. Digital competence is shaped not only by individual skills but also by management support, organisational practices and collegial support.

**Aim:**

To describe healthcare professionals’ assessments of socio‐organisational aspects associated with their digital competence development and to identify profiles of socio‐organisational support based on these assessments.

**Methods:**

A cross‐sectional study was conducted in 2024 in Estonia. The Aspects Associated with Digital Health Competence (DigiComInf) and Digital Health Competence (DigiHealthCom) instruments were applied. Healthcare professionals from different professional groups and clinical settings were recruited through convenience sampling. The K‐means cluster methodology was the main tool for analysing the data.

**Results:**

A total of 464 healthcare professionals were included in the analysis. Management and collegial support for digital competence development were rated moderate, whereas organisational practices were rated low. Three socio‐organisational support profiles were identified, with digital competence moderate across all but showing a clear gradient, highest in the High Support profile and lowest in the Low Support profile. The profiles also differed according to healthcare professionals’ personal, work‐related and technology training–related factors.

**Conclusions:**

Healthcare professionals reported different patterns of socio‐organisational support for digital competence development. The findings suggest that managerial engagement, organisational practices and collegial support may be relevant for digital competence development. Further research should examine managers’ perspectives and the relationship between managerial and professional views of these support structures.

**Implications for Nursing Management:**

The socio‐organisational support profiles identified in this study may serve as a practical framework for nurse managers to identify where managerial, organisational or collegial support for digital competence development could be strengthened. A coordinated organisational and policy‐level approach could support more consistent and systematic support for digital competence development across healthcare settings.

## 1. Introduction

The increasing integration of digital technologies into healthcare has changed professional practice and competence requirements for healthcare professionals [1]. Digital competence is now widely recognised as a prerequisite for the safe, effective and ethical use of digital technologies in healthcare delivery [1, 2], including diagnostics, treatment, patient monitoring, information management and communication [3, 4]. Digital competence includes the ability to use digital technologies effectively and responsibly, supported by technical and communication skills, knowledge of data security and ethics and a positive attitude towards digital tools [1, 5]. Beyond individual capabilities, digital technology use and competence development are related to broader social and organisational aspects of the workplace [5–7]. Social aspects include colleagues’ attitudes towards digital technologies, collegial influence and supportive workplace relationships [5, 7, 8]. Organisational aspects include management support, regular education and orientation, adequate technological resources and practices that facilitate competence development [5, 7, 8]. These social and organisational aspects are interconnected, with colleagues’ influence, management support and organisational practices jointly reflecting workplace context for digital competence development [1, 9]. Therefore, efforts to strengthen digital competence are unlikely to be effective if they focus solely on individual healthcare professionals without addressing the surrounding social and organisational context [10, 11].

From the perspective of managers, supporting and managing healthcare professionals’ digital competence development is an essential component of healthcare leadership in rapidly evolving healthcare systems [8, 12, 13]. Higher digital competence among healthcare professionals has been associated with active managerial support and training opportunities [6, 14]. Managers can facilitate the development of digital competence by allocating time for training, encouraging the use of digital tools and integrating digital competence objectives into everyday clinical workflows [5, 8]. Management‐supported organisational practices, including continuous education, clear guidelines and technical infrastructure, can strengthen staff confidence in using new technologies [1, 8]. Managers must also address insufficient equipment, inadequate infrastructure and limited training opportunities, which have been identified as major barriers to digital competence development [8, 15]. Healthcare professionals often lack sufficient training opportunities, and the available learning conditions may not align with individual learning needs [16, 17]. In addition to managerial and organisational factors, nurse managers can facilitate collegial support by promoting informal learning, experience sharing and peer mentoring among staff [6]. Positive social influence from colleagues is associated with higher digital competence and greater acceptance of digital solutions in clinical practice [6, 8].

Previous empirical research on digital competence in healthcare has largely focused on individual healthcare professionals and differences in their competence levels [18, 19] or grouping healthcare professionals according to their digital competence levels while examining organisational factors as secondary influences [6, 20]. While these studies provide valuable insight into differences in digital competence levels, they prioritise individual outcomes and may overlook the complex work environments in which healthcare professionals operate [21]. Focusing directly on socio‐organisational support, therefore, offers a more context‐sensitive understanding of how clinical and organisational environments support or limit the development of digital competence. Healthcare organisations are complex systems in which structures, leadership and professional relationships continuously interact and shape outcomes rather than functioning in separate [21]. Identifying distinct socio‐organisational profiles through person‐centred analyses generates actionable knowledge for healthcare managers and policymakers aiming to strengthen digital readiness across sectors, levels of care and professional groups [22].

The aim of this study was to describe healthcare professionals’ assessments of socio‐organisational aspects associated with their digital competence development and to identify profiles of socio‐organisational support based on these assessments.

We had three research questions.

1. How do healthcare professionals assess management support, organisational practices and colleague influence associated with their digital competence development?
2. What socio‐organisational profiles can be identified among healthcare professionals based on assessments of management support, organisational practices and peer influence?
3. Which professional and organisational background factors are associated with these socio‐organisational profiles?

## 2. Materials and Methods

### 2.1. Study Design

We used a cross‐sectional study design [23] with self‐reported online questionnaires for healthcare professionals working in Estonia in 2024. The data were analysed using descriptive statistical methods and cluster analysis. The study was reported in accordance with the Strengthening the Reporting of Observational Studies in Epidemiology (STROBE) guidelines [24].

### 2.2. Research Environment and Target Group

The study was conducted in Estonia, which is recognised for its advanced digital health infrastructure and nationwide e‐health initiatives [25, 26]. Our target group consisted of healthcare professionals working in different clinical settings. Healthcare services in Estonia are delivered by both public and private providers and include primary care, specialist and hospital care, nursing care and other healthcare services. Healthcare is predominantly publicly financed through the Estonian Health Insurance Fund. Healthcare organisations vary considerably in size and function, ranging from small primary and specialist care providers to large hospitals providing highly specialised care. In 2025, approximately 1500 healthcare service providers operated in Estonia, and about 75% employed fewer than ten healthcare professionals, whereas only 21 employed 250 or more [27]. Altogether, approximately 16,000 higher education–qualified healthcare professionals are employed in healthcare institutions in Estonia [28]. Healthcare professionals working in different clinical settings and from various professional groups were invited to participate in the study, including nurses, midwives, physicians, physiotherapists, radiographers, bioanalysts and occupational therapists.

### 2.3. Instruments

Data were collected with two self‐reported instruments, the *Aspects Associated with Digital Health Competence* (DigiComInf) and *Digital Health Competence* (DigiHealthCom) [1], and a background questionnaire. The background questions were focused on sociodemographic information and work‐related characteristics. In addition, two of the ten items from the *Shinners Artificial Intelligence Perception* (*SHAIP*) instrument [29] were used as background questions to assess whether participants perceived they had received adequate role‐specific AI training and that an ethical framework for AI use was present in their workplace. The rest of the SHAIP results have been reported elsewhere.

*DigiComInf* measures socio‐organisational aspects associated with healthcare professionals’ digital competence with 15 items divided into three factors: support from management (6 items), organisational practices as part of digital competence development (4 items) and colleagues’ adoption and influence (5 items) [1]. Management support includes leadership, feedback, managerial example and support for training and digital implementation. Organisational practices cover education, orientation and needs‐based competence development. Colleagues’ adoption and influence comprise colleagues’ attitudes, digital competence and willingness to use and develop digital solutions [1, 9]. *DigiHealthCom* measures healthcare professional’s digital competence through 42 items encompassing five dimensions: human‐centred remote counselling competence (16 items), digital solutions as part of work (9 items), information and communication technology (ICT) competence (5 items), competence in utilizing and evaluating digital solutions (8 items) and ethical competence related to digital solutions (4 items) [1]. For both instruments, items are assessed on a 4‐point Likert scale (1 = completely disagree to 4 = completely agree). The *SHAIP* measures healthcare professionals’ perceptions of AI in relation to their professional role and preparedness to use AI with 10 items on a five‐point Likert scale (completely disagree to completely agree) [29]. All three instruments have demonstrated acceptable validity and reliability in previous studies [1, 9, 18, 29].

The original instruments in English were translated and culturally adapted for the Estonian context using a five‐step process [30]. First, an educated translator conducted the forward translation. Second, the research teams reviewed the translations. Third, another educated translator performed the back‐translation, which was then compared with the original version by a native English speaker. Fourth, eight healthcare professionals assessed the relevance and clarity of instruments. Based on their assessments, the item‐level content validity index (I‐CVI) and scale‐level content validity indices, universal agreement method (S‐CVI/UA) and the average method (S‐CVI/AVE), were calculated [31]. For *DigiComInf,* all item‐level and scale‐level content validity scores reached the maximum value of 1.00, indicating complete agreement among the experts. For *DigiHealthCom*, the experts gave high ratings for relevance (I‐CVI = 0.88–1.00; S‐CVI/AVE = 0.99; S‐CVI/UA = 0.95). Ratings for clarity were slightly lower (I‐CVI = 0.63–1.00; S‐CVI/AVE = 0.97; S‐CVI/UA = 0.83). Two items with lower clarity ratings were revised and then re‐evaluated by three experts, and both received the maximum I‐CVI score of 1.00. Finally, the instruments were piloted with four master’s students in nursing science to test clarity, completion time and usability in the Research Electronic Data Capture (REDCap) application. REDCap is a secure web‐based application for data collection and management [32]. No changes were made, and no items were removed after the translation and cultural adaptation process. The internal reliability of the instruments was assessed using the Cronbach’s α coefficient. All instruments showed good internal consistency (*DigiComInf: α* = 0.93, subscales *α* = 0.86–0.91; *DigiHealthCom: α* = 0.96, subscales *α* = 0.81–0.96; *SHAIP*: *α* = 0.84, subscales *α* = 0.68–0.87).

### 2.4. Recruitment and Data Collection

Convenience sampling [33] was used to recruit healthcare professionals working in Estonia. Invitations were distributed in collaboration with 55 healthcare professional associations to reach professionals from diverse institutions, settings and occupation groups. According to publicly available data, these professional associations collectively represent approximately 18,000 members, although membership overlap is common and healthcare professionals often belong to more than one association. We asked professional associations to forward the invitation and study details to their members via mailing lists and social media channels. Additionally, we requested that the invitation to the study be shared on the social media pages and mailing lists of two higher education institutions in healthcare. The data were collected from the 8th of March to the 31st of May 2024. Two reminders were sent after 3‐4‐week intervals. Due to overlapping membership in associations and multiple recruitment channels, the number of healthcare professionals who received the invitation could not be determined, and therefore, the response rate could not be calculated.

### 2.5. Data Analysis

IBM SPSS Statistics (Released 2023. Version 29.0.2.0, Armonk, NY: IBM Corp) and R Statistical Software (Released 2026. Version 4.6.0, R Core Team) were used to analyse the data. Cluster validation in R used libraries *cluster* (Version 2.1.8.2) [34], *fpc* (Version 2.2‐15) [35] and *clusterCrit* (Version 1.3.0) [36] packages. Of the 507 healthcare professionals who completed the questionnaire, 464 provided complete responses to all 15 items of the *DigiComInf* instrument and were included in the study. Some background variables had missing values, which were not imputed. Descriptive statistical data were presented as frequencies and percentages, while mean scores and standard deviations were reported for continuous variables. Items from the *DigiComInf* factors were averaged for further analysis. We used the same cut‐off values for the level as the original authors of the *DigiComInf*, where ≤ 2.49 indicates low, 2.5–3.49 moderate and ≥ 3.50 high level support, according to the meaning of the Likert scale used with the items [6].

To identify distinct patterns in socio‐organisational support aspects associated with digital competence development, we applied an unsupervised machine learning approach, K‐means cluster analysis [37], using the standardised mean scores (*z*‐scores) for *DigiComInf*’s three factors: support from management, organisational practices and colleagues’ adoption and influence. The optimal number of clusters was determined by evaluating the elbow method, silhouette scores [38], the gap statistic [39] and the interpretability of the cluster solutions [40]. Although some measures favoured two clusters, this distinction was only between broadly higher and lower support. The elbow and gap criteria, together with interpretability, supported a three‐cluster model (silhouette score = 0.328). Cluster validation results are presented in Supporting Information 1. The K‐means algorithm was run with 500 random starts, and cluster stability was confirmed by nonparametric bootstrap resampling, with agreement of cluster membership quantified using the Jaccard coefficient [41] (mean Jaccard similarity ≥ 0.93 for all clusters). The modest silhouette score (0.328) suggests that the profiles overlap and reflect different levels of support rather than completely separate groups. However, the agreement between the validation measures and the high bootstrap stability indicates that the three‐cluster solution is reliable. The solution was also cross‐checked in IBM SPSS Statistics, which produced consistent results. The clusters were interpreted as three distinct profiles of socio‐organisational support, reflecting the levels of socio‐organisational support within each group.

We also examined whether sociodemographic, organisational, training for digital competence and professional experience–related variables differed across profiles. These comparisons were based on available data for each variable, so sample sizes varied due to missing responses. Two items derived from the SHAIP instrument, representing receiving adequate role‐specific AI training and the presence of an ethical framework for AI use in the workplace, were recoded prior to analysis into binary variables (yes/no). Dichotomisation was chosen because the items were used as indicators of the perceived presence or absence of these organisational conditions rather than as measures of the degree of agreement. Responses of ‘agree’ and ‘completely agree’ were classified as affirmative and coded as ‘yes’, whereas ‘unsure’, ‘disagree’ and ‘strongly disagree” were classified as nonaffirmative and coded as ‘no’. As a sensitivity analysis, the original five‐category responses were also compared across profiles using the Kruskal–Wallis test. The results were consistent with the dichotomised analysis, indicating the recoding did not weaken the observed associations. Profiles were further characterised using the mean score of the *DigiHealthCom* instrument to provide an overall level of digital competence. The classification of competence levels followed the cut‐off values defined by the original authors of the *DigiHealthCom*, with scores ≤ 2.49 representing low, 2.50–3.49 moderate and ≥ 3.50 high competence [1]. Pearson’s chi‐square test was used to compare categorical variables across profiles, with Cramér’s V reported as the effect size. For continuous variables, normality was assessed using the Shapiro–Wilk test and Q–Q plots, and homogeneity of variance was assessed using Levene’s test. One‐way analysis of variance (ANOVA) with post hoc comparisons (Tukey’s HSD) was used when the assumption of homogeneity of variance was met. Otherwise, Welch’s ANOVA with Games–Howell post hoc comparisons was applied. Eta‐squared (*η* ^2^) was reported as the effect size. The level of statistical significance was set at *α* = 0.05 in all tests.

### 2.6. Ethical Considerations

The Research Ethics Committee (blinded for review) approved the study (protocol number 348/T‐18). Participants were informed about the aim of the study, the voluntary and anonymous nature of participation [42] and the principles of data protection and confidentiality [43]. Informed consent was requested at the beginning of the questionnaire. Before submitting their responses, participants had the right to withdraw from the study without any consequences. Permission to translate and adapt the instruments for the Estonian context and to use them in this study was obtained from the original instrument authors.

## 3. Results

### 3.1. Background Data of Respondents

A total of 464 healthcare professionals with complete *DigiComInf* data were included in the analysis. Most of them were female (90.3%). The mean age was 44.4 years (SD = 12.6), and the average work experience in healthcare was 19.5 years (SD = 13.6; median = 17.0). Half of the participants (54.2%) were nurses and midwives. Two‐thirds of participants (66.8%) had received digital competence training at work, while almost half (46.8%) reported developing digital competence on their own time outside of working hours. Adequate AI training specific to the professional role was reported by 15.0% of respondents (Table 1).

**TABLE 1:** Background characteristics of healthcare professionals (*n* = 464).

	*n*	%
Sociodemographic characteristics		
Sex (*n* = 453)		
Female	409	90.3
Male	43	9.5
Other	1	0.2
Age in years (n = 463)	*M* = 44.4 (SD = 12.6)	
Education (n = 464)		
Vocational or bachelor	241	51.9
Master or doctoral	223	48.1
Profession (n = 463)		
Nurse or midwife	251	54.2
Physician	132	28.5
Other professional^1^	80	17.3
Contract type (n = 461)		
Full‐time	368	79.8
Part‐time	93	20.2
Work experience in healthcare (n = 462)	*M* = 19.5 years (SD = 13.6); Md = 17.0	
Organisation characteristics		
Organisation type (n = 464)		
Public hospital	315	67.9
Family or primary health centre	84	18.1
Private clinic or company	36	7.8
Other^2^	29	6.3
Healthcare setting (n = 464)		
Inpatient care	188	40.5
Outpatient care	183	39.4
Community health services (day hospital, home care, emergency services)	20	4.3
Diagnostics and research	42	9.1
Other^3^	31	6.7
Digital competence training and professional experience (n = 464)		
Digital competence training at work	310	66.8
Digital competence development in own time	217	46.8
Involvement in digital solution development	152	32.8
Been educator in digital health training	30	6.5
AI training and ethical framework for AI use at work		
Adequate AI training specific to professional role (*n* = 460)	69	15.0
Ethical framework is in place for AI use at work (*n* = 458)	59	12.9

Abbreviations: M, mean; Md, median; SD, standard deviation.

^1^E.g., radiographer, bioanalyst, physiotherapist, occupational therapist or other healthcare professional.

^2^E.g., homecare institution, social care institution and special welfare institution.

^3^E.g., online services, administration and social services.

### 3.2. Assessment of Socio‐Organisational Aspects Related to Digital Competence Development

Healthcare professionals assessed management support (*M* = 2.7, SD = 0.86) and colleagues’ adaption and influence (*M* = 2.9, SD = 0.65) at a moderate level, while organisational practices were rated low (*M* = 2.4, SD = 0.84). Most healthcare professionals (78.9%) agreed that their manager supports the implementation of digital solutions, and top management supports digital update (74.8%). Additionally, almost half (45.3%) agreed that managers lead their digital competence development, and 39.0% that managers provide feedback on their digital competence development. Less than half of healthcare professionals (43.1%) agreed that digital competence development is planned according to their individual needs, and 41.2% agreed that orientation to digital solutions is systematic within their working unit. Most professionals (88.6%) agreed that colleagues do not negatively influence their digital competence development, while 55.0% agreed that colleagues are eager to develop their work using digital solutions (Table 2).

**TABLE 2:** Assessment of socio‐organisational aspects related to digital competence development (Likert scale 1–4).

Factor/item	Agree		Disagree		M (SD)	CI 95%
	*n*	%	*n*	%		
Management support (*n* = 464)					2.70 (0.86)	2.62–2.78
Manager supports digital solution implementation	366	78.9	98	21.1	3.11 (0.95)	3.03–3.20
Top management supports digital uptake	347	74.8	117	25.2	3.02 (0.94)	2.93–3.10
Manager’s example supports digital competence	324	69.8	140	30.2	2.87 (1.04)	2.77–2.96
Manager supports digital competence training	292	62.9	172	37.1	2.74 (1.10)	2.64–2.84
Manager leads digital competence development	210	45.3	254	54.7	2.33 (1.06)	2.23–2.42
Manager provides feedback on digital competence	181	39.0	283	61.0	2.15 (1.07)	2.05–2.25
Organisational practices (*n* = 464)					2.39 (0.84)	2.32–2.47
Sufficient education about digital solutions at work	260	56.0	204	44.0	2.57 (0.94)	2.49–2.66
Organisational practices support digital competence	250	53.9	214	46.1	2.49 (0.96)	2.40–2.58
Digital competence development based on individual needs	200	43.1	264	56.9	2.27 (1.00)	2.18–2.36
Systematic orientation to digital solutions	191	41.2	273	58.8	2.24 (0.97)	2.15–2.33
Colleagues’ adaption and influence (*n* = 464)					2.87 (0.65)	2.81–2.93
Colleagues do not hinder digital competence development	411	88.6	53	11.4	3.36 (0.76)	3.29–3.43
Positive perceptions of digital implementation at work	318	68.5	146	31.5	2.84 (0.80)	2.77–2.91
Colleagues have good digital competence level	314	67.7	150	32.3	2.83 (0.83)	2.75–2.90
Colleagues are not reluctant to use digital solutions	297	64.0	167	36.0	2.74 (0.81)	2.67–2.82
Colleagues are eager to develop work with digital solutions	255	55.0	209	45.0	2.56 (0.87)	2.49–2.64

*Note:* Likert responses were dichotomised: agree (strongly agree/agree) and disagree (disagree/strongly disagree).

Abbreviations: M, mean; SD, standard deviation.

### 3.3. Identified Healthcare Professionals’ Socio‐Organisational Support Profiles

Three distinct profiles of socio‐organisational support for digital competence development were identified among 464 healthcare professionals. These profiles reflected differing levels of management support, organisational practices and colleagues’ influence (Table 3). The first profile, named *High Support*, comprised 105 respondents (22.6%), characterised by high levels of management support (*M* = 3.63, SD = 0.40) and colleague adaption and influence (*M* = 3.62, SD = 0.39) and moderate level of organisational practices (*M* = 3.34, SD = 0.51). The second profile, named *Moderate Support*, consisted of 211 respondents (45.5%) and displayed moderate levels of management support (*M* = 2.90, SD = 0.49), organisational practices (*M* = 2.55, SD = 0.47) and colleagues’ adaption and influence (*M* = 2.83, SD = 0.40). The third profile, named *Low Support*, included 148 respondents (31.9%) and showed a low level of support across all socio‐organisational factors: management support (*M* = 1.76, SD = 0.53), organisational practices (*M* = 1.49, SD = 0.45) and colleagues’ adaption and influence (*M* = 2.37, SD = 0.59).

**TABLE 3:** Socioorganisational support profiles based on mean scores of DigiComInf (Likert scale 1–4).

Factor	Profile 1		Profile 2		Profile 3	
	High Support (*n* = 105; 22.6%)		Moderate Support (*n* = 211; 45.5%)		Low Support (*n* = 148; 31.9%)	
	*M* (SD)	*z*	*M* (SD)	*z*	*M* (SD)	*z*
Management support	3.63 (0.40)	+1.08	2.90 (0.49)	+0.24	1.76 (0.53)	−1.10
Organisational practices	3.34 (0.51)	+1.13	2.55 (0.47)	+0.19	1.49 (0.45)	−1.08
Colleague adaption and influence	3.62 (0.39)	+1.16	2.83 (0.40)	−0.05	2.37 (0.59)	−0.76

*Note:* Socio‐organisational support classification: ≤ 2.49 low, 2.5–3.49 moderate and ≥ 3.50 high.

Abbreviations: M, mean; SD, standard deviation; *z*, standardised cluster centroid (*z*‐score).

Participants in the first profile, *High Support*, were the oldest (*M* = 47.8 years; SD = 13.4), compared with other profiles (*p* = 0.005). They also had the longest work experience in healthcare (*M* = 23.6 years; SD = 14.6; *p* < 0.001). No significant differences were observed with education level, profession or contract type. Organisation type and healthcare setting differed significantly across all profiles (both *p* < 0.001), with this profile somewhat more common in private clinics and primary healthcare organisations, as well as in outpatient care settings. Respondents in this profile demonstrated a moderate level of digital competence (*M* = 3.46; SD = 0.39), which was higher than that in other profiles (*p* < 0.001). They more frequently reported having received digital competence training at work, being involved in the development of digital solutions and were more likely to report adequate AI training (all *p* < 0.001). The presence of an ethical framework for AI use in the workplace was more common in this profile than in the other profiles (*p* < 0.001). No differences were observed in the development of digital competence on one’s own time across the profiles (Table 4).

**TABLE 4:** Socio‐organisational support profiles and background characteristics (*n* = 464).

Variable	Profile 1		Profile 2		Profile 3		*p* value		
	High Support		Moderate Support		Low Support				
	*n*	%	*n*	%	*n*	%			
Sociodemographic characteristics									
Age in years (*n* = 463)									
Mean (SD); Min–Max	47.8^a^ (13.4); 22–84		43.9^b^ (12.3); 22–74		42.7^b^ (11.9); 23–75		**0.005** ^∗∗^	*η* ^2^ = 0.023	
Education (*n* = 464)									
Vocational or bachelor	57	54.3	118	55.9	66	44.6	0.092^†^	*V* = 0.101	
Master or doctoral	48	45.7	93	44.1	82	55.4			
Profession (*n* = 463)									
Nurse or midwife	59	56.2	118	56.2	74	50.0	0.806^†^	*V* = 0.042	
Physician	28	26.7	58	27.6	46	31.1			
Other professional^1^	18	17.1	34	16.2	28	18.9			
Contract type (*n* = 461)									
Full time	83	79.8	168	80.4	117	79.1	0.954^†^	*V* = 0.014	
Part time	21	20.2	41	19.6	31	20.9			
Work experience in healthcare (*n* = 462)									
Mean (SD); Min–Max	23.6^a^ (14.6); 0–65		19.4^b^ (13.3); 0–53		16.6^b^ (12.9); 0–55		**< 0.001** ^∗∗^	*η* ^2^ = 0.035	
Organisation characteristics									
Organisation type (*n* = 464)									
Public hospital	53	50.5	156	73.9	106	71.6	**< 0.001** ^†^	*V* = 0.180	
Private clinic or company	32	30.5	32	15.2	20	13.5			
Family or primary health centre	16	15.2	9	4.3	11	7.4			
Other^2^	4	3.8	14	6.6	11	7.4			
Healthcare setting (*n* = 464)									
Inpatient care	27	25.7	88	41.7	73	49.3	**< 0.001** ^†^	*V* = 0.172	
Outpatient care	57	54.3	76	36.0	50	33.8			
Community health services	1	1.0	9	4.3	10	6.8			
Diagnostics and research	9	8.6	24	11.4	9	6.1			
Other^3^	11	10.5	14	6.6	6	4.1			
Digital competence (*n* = 464)									
Mean (SD); Min–Max	3.46^a^ (0.39); 2.25–4.0		3.20^b^ (0.46); 1.71–4.0		3.00^c^ (0.61); 1.36–4.0		**< 0.001** ^∗^		*η* ^2^ = 0.099
Digital competence training and professional experience									
Digital competence training at work (*n* = 464)	83	79.0	153	72.5	74	50.0	**< 0.001** ^†^		*V* = 0.250
Digital competence development in own time (*n* = 464)	56	53.3	100	47.4	61	41.2	0.159^†^		*V* = 0.089
Involvement in digital solution development (*n* = 464)	43	41.0	77	36.5	32	21.6	**0.002** ^†^		*V* = 0.167
Been educator in digital health training (*n* = 464)	11	10.5	13	6.2	6	4.1	0.120^†^		*V* = 0.096
AI training and ethical framework for AI use at work									
Adequate AI training (*n* = 460)	26	24.8	34	16.3	9	6.2	**< 0.001** ^†^		*V* = 0.193
Ethical framework in place for AI use at work (*n* = 458)	29	27.9	25	11.9	5	3.5	**< 0.001** ^†^		*V* = 0.266

*Note:* Superscript letters (a, b, c) indicate significant differences between profiles based on Tukey HSD or Games–Howell post hoc tests. Means sharing the same letter do not differ significantly. Bold *p* values indicate statistically significant results (*p* < 0.05).

^†^Pearson’s chi‐square test (effect size Cramér’s V).

^∗∗^One‐way ANOVA (effect size eta‐squared (*η* ^2^), Tukey HSD post hoc).

^∗^Welch’s ANOVA (effect size eta‐squared (*η* ^2^), Games–Howell post hoc).

^1^E.g., radiographer, bioanalyst, physiotherapist, occupational therapist or other healthcare professional.

^2^E.g., homecare institution, social care institution and special welfare institution.

^3^E.g., online services, administration and social service.

The second profile, named *Moderate Support*, consisted of younger professionals than those in the first profile (*p* = 0.005), but it was like the third profile (*M* = 43.9 years; SD = 12.3). They had less work experience in healthcare than in the first profile but more than those in the third profile (*M* = 19.4 years; SD = 13.1; *p* < 0.001). This profile was more frequently represented in public hospitals. Professionals in this profile are distributed fairly evenly between inpatient and outpatient care. Compared to other profiles, working in settings related to diagnostics and research was more likely. Respondents in this profile demonstrated a moderate level of digital competence (*M* = 3.20; SD = 0.46), which was lower than that in the first profile but higher than that in the third profile (*p* < 0.001). Digital competence training, involvement in digital development, adequate AI training and the presence of an ethical framework for AI use were intermediate compared with the other profiles (Table 4).

Participants in the third profile, *Low Support*, were younger (*M* = 42.7 years; SD = 11.9; *p* < 0.005) and had less work experience (*M* = 16.6 years; SD = 12.9; *p* < 0.001) than in the first profile, but were like the second profile. This profile was more frequently represented in public hospitals, although differences were modest. Participants in this profile worked more frequently in inpatient care. This profile also showed a moderate level of digital competence (*M* = 3.00; SD = 0.61), but it was lower than that in other profiles (*p* < 0.001). Respondents in this profile least frequently reported workplace‐based digital competence training and involvement in digital solution development. Adequate AI training and the presence of an ethical framework for AI use were rarely reported (Table 4).

## 4. Discussion

This study found variation in healthcare professionals’ perceived socio‐organisational support for digital competence development. Management and collegial support were rated moderate, while organisational practices related to the systematic planning and implementation of digital competence development were rated low. We also identified three distinct socio‐organisational support profiles, named as *High Support, Moderate Support and Low Support.* Each profile reflects different configurations of socio‐organisational support. Self‐reported digital competence was moderate in all three profiles but differed across them, with the highest mean in the *High Support* profile and the lowest in the *Low Support* profile. The profiles also differed in background characteristics, such as healthcare professionals’ age, work experience, organisation type and healthcare setting. They also differed in digital and AI‐related factors, including digital competence and AI‐related training, involvement in the development of digital solutions and perceived presence of an ethical framework for AI use in the workplace.

Healthcare professionals assessed management support and colleagues’ influence to be higher than organisational practices in developing digital competence. This suggests that supportive interpersonal environments may exist even when structured organisational practices, such as systematic training, clear implementation planning and adequate resource allocation, remain insufficient. A recent systematic review concluded that initiatives to strengthen digital competence should address social and environmental conditions in addition to individual capabilities [44]. Evidence concerning healthcare professionals’ digital competence and associated factors also emphasises the importance of educational opportunities and work environments that enable professionals to apply their competence [7]. In the Estonian context, these findings are particularly noteworthy given the country’s high level of digital maturity and advanced e‐health infrastructure (e.g., health portal since 2008) [25, 26]. The results indicate that national technological advancement does not automatically translate into structured workplace practices that support digital competence development. Managers may therefore play an important role in bridging this gap by strengthening organisational practices, facilitating dialogue with staff and supporting continuous learning [45]. Managers’ own digital competence may also influence how effectively they can support and organise the development of digital competence among their staff [11]. Further research is needed to understand how managers themselves perceive and support the development of digital competence within healthcare organisations.

The three socio‐organisational support profiles show that healthcare professionals perceived different combinations of managerial, organisational and collegial support. The *High Support* profile represents environments characterised by strong managerial support and positive collegial influence, while organisational practices are at a moderate level. The *Moderate Support* profile reflects contexts in which management support, organisational practices and colleagues’ influence are all at moderate levels, indicating a relatively balanced but not strongly developed environment for digital competence development. The *Low Support* profile represents environments with limited support across all three socio‐organisational support dimensions. These patterns are consistent with the view of healthcare organisations as interconnected systems in which managerial practices, structures and professional interactions shape workplace conditions [21]. A lack of organisational support and training has been identified as a key barrier to digital readiness in healthcare [8]. When support is limited, professionals may have fewer opportunities to build competence [12], which can affect both the use of digital tools and overall readiness for digital transformation.

The *High Support* profile was associated with both higher self‐reported digital competence and greater access to workplace‐based digital and AI‐related training than the other profiles. This is consistent with previous research highlighting the role of organisational context and management in shaping learning opportunities [8]. Education has been identified as an important aspect for strengthening digital health competence [44]. Nevertheless, digital competence remained within the moderate range in all profiles, and even the *High Support* profile only approached, without reaching the predefined threshold for high competence. Organisational practices also remained at a moderate level in the *High Support* profile, despite high levels of management support and colleagues’ influence. Thus, strong managerial and collegial support may not be sufficient on its own to achieve high digital competence, and more systematic organisational practices may also be important. At the same time, digital competence is likely shaped by factors beyond organisational environment, including individual experience, opportunities to use technologies in practice and engagement in learning [7]. Digital competence development during personal time did not differ between the profiles. By contrast, workplace‐based training was more frequent in the *High Support* and *Moderate Support* profiles than in the *Low Support* profile. This finding suggests that supportive socio‐organisational environments may be more closely connected to workplace learning opportunities than to competence development undertaken on professionals’ own initiative. The profiles also differed by age and work experience. Older and more experienced healthcare professionals were more often represented in the *High Support* profile. This finding differs from evidence associating younger age with stronger self‐perceived digital competence [7]. However, the results are not directly contradictory, as the present profiles reflect perceived socio‐organisational support rather than digital competence alone. Older and more experienced professionals may have stronger professional networks, greater access to organisational resources or increased involvement in workplace development activities over time.

The profiles were further associated with organisational types and healthcare settings. *Moderate Support* and *Low Support* profiles were more commonly presented among professionals working in public hospitals and inpatient care, whereas the *High Support* profile was more common in outpatient and nonhospital settings. Work setting has also been identified as a factor associated with digital health competence in previous studies [7]. The findings suggest that the organisational context may be relevant to how healthcare professionals perceive socio‐organisational support for the development of digital competence. One possible explanation may relate to differences in organisational structure and complexity across healthcare settings. For example, larger hospitals may face greater challenges in coordinating and implementing support for digital competence development consistently across units. However, as organisational size and complexity were not assessed in this study, this needs further investigation. The relatively limited availability of AI‐related training and ethical guidance across profiles may indicate that organisational preparedness for AI implementation has not kept pace with technological advancement in healthcare.

These socio‐organisational support profiles reflect current conditions within evolving organisational contexts shaped by ongoing changes in professional roles [46, 47], organisational structures [48] and healthcare services [49]. As professionals gain experience, participate in continuing education training or move between roles and healthcare settings, their support needs may change. Likewise, organisational reforms, management development or strengthened strategic planning can reshape support structures over time. The profiles, therefore, provide a way to understand how digital competence support is distributed at a given moment, while also offering a basis for monitoring change and guiding workforce and organisational development.

### 4.1. Implications for Healthcare Management and Policy

Digital competence development should be considered both an individual and an organisational responsibility. Three socio‐organisational support profiles identified in this study may serve as a practical framework for healthcare managers to identify where managerial, organisational or collegial support could be strengthened. However, the profiles should not be used to classify individual employees or as evidence that any form of support causes higher competence. Healthcare settings with lower perceived support may benefit from more structured orientation, clearer guidelines and increased managerial involvement, whereas settings with moderate support may benefit from strengthened feedback mechanisms and opportunities for peer learning and better integration of competence development into everyday work.

The variation in perceived support across healthcare settings suggests that organisational initiatives should be supported by coordinated policies. This need is not unique to Estonia, as continuing education in digital competence differs across EU countries in its organisation, funding and accreditation [50]. There is a need for uniform and regularly updated areas of digital competence for healthcare professionals, defined at the national and EU levels. Some European countries have already introduced national strategies and defined areas of digital competence for healthcare professionals [51]. A nationally adopted, healthcare‐wide digital competence framework, which, to our knowledge, is currently lacking in Estonia, could provide shared and regularly updated goals for workforce planning, professional standards and continuing education. However, a framework alone would not ensure the development of digital competence. Its practical value would depend on organisational support, including managers who prioritise learning, adequate resources and training arrangements and collegial support for applying digital skills in practice.

### 4.2. Strengths and Limitations

The strengths of this study are a profile‐based analysis of socio‐organisational support for digital competence development, moving beyond average‐level descriptions. By integrating managerial, organisational and collegial dimensions of support alongside background characteristics and digital competence level, the study offers a comprehensive view of how digital competence is embedded within professional and organisational contexts. The sample broadly reflected the structure of the Estonian healthcare workforce in terms of age, sex, professional groups and workplace settings [28, 52]. The predominance of female participants, nurses and hospital employees was consistent with national workforce characteristics, supporting the relevance of the findings. However, because participation was voluntary, some self‐selection bias cannot be excluded.

At the same time, several limitations should be acknowledged. The cross‐sectional design limits causal inference, and the direction of the relationship between socio‐organisational support and digital competence cannot be determined [53]. A longitudinal research design would be needed to examine how professionals may transition between support profiles over time and how changes in organisational structures influence competence development. Self‐reported measures may introduce response bias and may not fully reflect objectively assessed organisational practices or digital competence [54]. Moreover, the identified profiles are sample‐dependent [40] and reflect a specific healthcare context, which may limit generalisability to other systems with different leadership models or levels of digital maturity. The response rate could not be determined because the number of unique healthcare professionals who received the study invitation was unknown due to the use of multiple recruitment channels. As data were collected through an online questionnaire, healthcare professionals with lower levels of digital competence may have been underrepresented in the sample, potentially introducing digital divide bias. Finally, the study did not include detailed organisational characteristics, such as unit size, organisational performance indicators or the managerial level assessed, nor objective indicators of organisational digital strategy or leadership behaviour. These factors could provide deeper insight into the mechanisms underlying the observed differences between profiles.

## 5. Conclusions

Digital competence development among healthcare professionals was associated with different patterns of socio‐organisational support. The findings suggest that management support, organisational practices and supportive workplace cultures are relevant to digital competence development. Even in a digitally advanced healthcare system, organisational support for digital competence development may remain uneven, suggesting that technological maturity alone may not ensure consistent support. Coordinated organisational and policy‐level efforts may help to promote equitable and systematic digital competence development across healthcare settings. Further research is needed to examine how managers perceive and shape these support structures, and how differences between managerial and professional perspectives can be addressed.

## Author Contributions

Janne Kommusaar: conceptualisation, methodology, validation, formal analysis, investigation, data curation, writing–original draft and visualisation. Laura‐Maria Peltonen: conceptualisation, methodology, investigation, writing–review and editing and supervision. Kadri Suija: conceptualisation, methodology, investigation, data curation, writing–review and editing and supervision. Erika Jarva: conceptualisation, methodology and writing–review and editing. Kristina Mikkonen: conceptualisation, methodology, writing–review and editing and project administration. Lauri Sillanmäki: methodology, validation and writing–review and editing. Mari Kangasniemi: conceptualisation, methodology, investigation, writing–review and editing, supervision and project administration.

## Funding

This research received no external funding.

## Conflicts of Interest

The authors declare no conflicts of interest.

## Supporting Information

Additional supporting information can be found online in the Supporting Information section.

## Supporting information


**Supporting Information 1** Supporting File 1 contains the cluster validation analyses, including validity indices and bootstrap stability for the candidate cluster solutions, as well as the elbow and gap‐statistic plots used to determine the number of clusters.

**Supporting Information 2** STROBE Statement—Checklist of items that should be included in reports of *cross-sectional studies*.

## Data Availability

The data that support the findings of this study are not publicly available due to ethical and data protection considerations.
