# Supplementary material for: Socio‐Organisational Aspects Related to Digital Competence Development Among Healthcare Professionals: A Cross‐Sectional Study With Cluster Analysis
Source: J Nurs Manag. 2026 Nov 3;2026:4368819. doi: 10.1155/jonm/4368819 (PMC13729262; doi:10.1155/jonm/4368819)
Supplement: Supplementary file 2 [file JONM-2026-4368819-s001.docx]

**Appendix 1**

**Table 1. Comparison of candidate solutions**

| **Clusters** | **WSS** | **WSS reduction** | **Silhouette** | **Calinski–Harabasz** | **Davies–Bouldin** | **Dunn** |
| --- | --- | --- | --- | --- | --- | --- |
| 2 | 714.96 | - | 0.395 | 435.562 | 0.948 | 0.042 |
| 3 | 534.13 | 25.3% | 0.328 | 368.915 | 1.042 | 0.040 |
| 4 | 445.28 | 16.6% | 0.304 | 324.967 | 1.133 | 0.046 |
| 5 | 383.37 | 13.9% | 0.274 | 301.010 | 1.166 | 0.040 |

**Table 2. Bootstrap stability**

| Solution | Cluster | Mean Jaccard | Dissolutions |
| --- | --- | --- | --- |
| 2 clusters | 1 | 0.973 | 0 |
|  | 2 | 0.965 | 0 |
| 3 clusters | 1 | 0.938 | 0 |
|  | 2 | 0.943 | 0 |
|  | 3 | 0.930 | 0 |

**Figure 1. Elbow plot for K-means solutions**

**Figure 2. Gap-statistic plot for candidate cluster solutions**
